# Multi-morbidity and disability, findings from the KORA-Age study

**DOI:** 10.1186/1753-6561-7-S4-S10

**Published:** 2013-08-23

**Authors:** Eva Grill, Angela Döring, Margit Heier, Rolf Holle, Karl-Heinz Ladwig, Birgit Linkohr, Christa Meisinger, Andreas Mielck, Holger Schulz, Barbara Thorand, Annette Peters

**Affiliations:** 1 Institute for Medical Information Processing, Biometry and Epidemiology, Ludwig-Maximilians-Universität München, Munich, Germany; 2 German Centre for Vertigo and Balance Disorders, Ludwig-Maximilians-Universität München, Munich, Germany; 3 Institute of Epidemiology I, Helmholtz Zentrum München, German Research Centre for Environmental Health, Neuherberg, Germany; 4 Institute of Epidemiology II, Helmholtz Zentrum München, German Research Centre for Environmental Health, Neuherberg, Germany; 5 Central Hospital Augsburg, MONICA/KORA Myocardial Infarction Registry, Augsburg, Germany; 6 Institute of Health Economics and Health Care Management, Helmholtz Zentrum München, German Research Centre for Environmental Health, Neuherberg, Germany

## 

KORA-Age is a multidisciplinary research consortium funded by the German Federal Ministry of Education and Research (FKZ 01ET1003). The objective of KORA-Age is to examine the prevalence and determinants of mortality, multi-morbidity, functioning and successful ageing in persons aged 65 years or older in a large cohort of randomly selected inhabitants of the region of Augsburg, Germany.

The KORA-Age project is carried out within the framework of KORA (Cooperative Health Research in the Region of Augsburg; http://www.helmholtz-muenchen.de/en/kora-en/index.html). The cohort includes all participants of the four cross-sectional MONICA/KORA Surveys S1-S4 conducted between 1984 and 2001 who were born in or before 1943, in total 9,197 persons. Within KORA-Age, a mortality follow-up was conducted in 2008 and 2011. In 2008/2009, a mailed questionnaire was answered by 4,565 individuals and a telephone interview with 4,127 individuals conducted. A physical examination was performed in a gender and age-stratified random sample of the cohort in 2009 in 1,079 persons. They were followed up three years later and a re-examination took place in 822 persons. Information was obtained from interviews, a self-assessment form and health examinations including morbidity, a comprehensive assessment on mental health, health services utilisation, medication and a complete characterisation of ageing-related biological parameters like cardiovascular and pulmonary function, tests of balance and stability, bone density, body composition and measures of multi-morbidity, frailty, disability and participation. In 2013 a group of older citizens will take part in qualitative focus groups on the lived experience of neighbourhood and environment to evaluate participation in-depth in the region of Augsburg. The sampling area was geocoded.

Major advantages of the study include the longitudinal design of the study with a wealth of data from previous surveys, and the comprehensive approach to functioning based on the model and framework of WHO’s International Classification of Functioning, Disability and Health (ICF). We propose this approach for integrating functioning into a comprehensive view of the prevalence and determinants of multi-morbidity, disability and successful ageing in interaction with personal and environmental factors.

